# Determination of chloroplast genome sequence of *Magnolia ofeliae* A. Vázquez & Cuevas (Magnoliaceae), an endangered Mexican Magnolia

**DOI:** 10.1080/23802359.2020.1840930

**Published:** 2020-12-24

**Authors:** Yoonju Yeo, Suhyun Park, J. Antonio Vázquez-García, Sangtae Kim

**Affiliations:** a Department of Biotechnology, Sungshin University, Seoul, Korea; b Department of Biology, Sungshin University, Seoul, Korea; c Departament of Botany and Zoology, Universidad de Guadalajara, Zapopan, Mexico

**Keywords:** Chloroplast genome, critically endangered species, *Magnolia ofeliae*, Magnoliaceae, Mexico

## Abstract

*Magnolia ofeliae* A. Vázquez & Cuevas, a plant species endemic to south Jalisco, Mexico, is a critically endangered (CR) species based on the IUCN Red List. In this study, we assembled its complete chloroplast (cp) genome. The total genome size of *M. ofeliae* was 159,839 bp including four subregions: a large single-copy (LSC) region of 88,027 bp and a small single-copy (SSC) region of 18,752 bp separated by a pair of identical inverted repeat regions (IRs) of 26,530 bp each. The GC content of the cp genome of *M. ofeliae* is 39.3%. The cp genome encoded a set of 113 genes, containing 79 protein-coding genes, 30 *tRNA* genes, and four *rRNA* genes. Phylogenetic analysis results that *M. ofeliae* is a sister to all other magnolias in the subfamily Magnolioideae.

An endemic species in Mexico, *Magnolia ofeliae* A. Vázquez & Cuevas is only distributed in the type locality (Vázquez-García et al. 2013). Despite intensive explorations in the Talpa de Allende region and elsewhere in Jalisco (Rivers et al. 2016), it is extremely rare. A second population (*ca*. 20 adult trees) was recently found in Santa Gertrudis, 20 km east of the type locality, with nearly 20 trees. Therefore, it is categorized as a critically endangered (CR) species in recently revised the red list of Magnoliaceae (Rivers et al. 2016; https://www.iucnredlist.org/species/67513575/67513783). This species is included in section *Talauma* in the recent classification systems of Magnoliaceae (Figlar and Nooteboom 2004, Wang et al. 2020) because the plant has circumscissile dehiscing folicetum (Vázquez-García et al. 2013).

Section *Talauma*, despite being the largest group in the family (*ca*. 100 spp.; Vázquez-García et al. 2016), is one of the less well-studied lineages of Magnoliaceae based on the recent classification system (Figlar and Nooteboom 2004). Up to date, there are 43 chloroplast (cp) genomes in the Magnoliaceae that have been registered in the organelle genome resources of the GenBank and none of them is from the section *Talauma*. In this study, we determined a complete sequence of the cp genome of *M. ofeliae* (GenBank accession no. MT522025), as a representative of the sect. *Talauma* in Magnoliaceae.

Leaf samples were collected from a tree in the Vallarta Botanica Garden (N20°27′55.14′′, W105°17′31.21′′), one of the *ex situ* conservation stations of Mexican magnolias. The tree was grown from seeds of the type locality, collected in June 2011 (*Vázquez-García 1968*, IBUG). A voucher specimen is deposited in the herbarium of the Sungshin University, Korea (*S. Kim 2019-0071*, SWU).

DNA extraction was performed using a commercial plant DNA extraction kit (Exgene^TM^; GeneAll, Seoul, Korea). The extracted DNA was sequenced using the Illumina platform at Macrogen Co. (Seoul, Korea). Paired-end sequencing generated a total of 21,451,116 raw reads after removing adapter sequences. The raw data is deposited in the GenBank SRA database (PRJNA666275). There was no need for the filtering of low-quality sequences because the Phred quality score of most of the reads was over 40 by eye examination of raw reads in Geneious Prime version 9.0.5 (Kearse et al. 2012).

The raw reads of *M. ofeliae* were mapped against reference sequences, multiple cp genomes previously published [*M. sinica* (NC_023241), *M. grandiflora* (NC_020318), and *M. tripetala* (*NC_024027*)], using Geneious Prime version 9.0.5 (Kearse et al. 2012). Border sequences of two inverted repeats and sequences of low coverage parts were checked manually, and the mapping for ambiguous regions is repeated. To verify the sequence, we tried de novo assembly using mapped reads on the reference using Geneious Prime version 9.0.5 (Kearse et al. 2012) with ‘medium sensitivity’ option, and the assembled result was exactly the same as mapped consensus. The completed sequence was annotated with the previously reported genome of *M. kobus* (GenBank Accession No. NC_023237) using Geneious Prime version 9.0.5 (Kearse et al. 2012).

The size of the cp genome of *M. ofeliae* is 159,839 bp, including four subregions: a large single-copy (LSC) region of 88,027 bp and a small single-copy (SSC) region of 18,752 bp separated by a pair of identical inverted repeat regions (IRs) of 26,530 bp each. The GC content of the cp genome of *M. ofeliae* is 39.3%. The cp genome encoded a set of 113 genes, containing 79 protein-coding genes, 30 tRNAs, and four rRNAs.

Phylogenetic analysis was performed using the cp genome sequence of *M. ofeliae* and 18 cp genomes that are from representatives of subfamily Magnolioideae and two outgroups (*L. chinense* and *L. tulipifera*) from subfamily Liriodendroideae (Figure 1). The alignment of these sequences was performed by the MAFFT module in Geneious Prime version 9.0.5 (Kearse et al. 2012). A maximum likelihood analysis has performed using IQ-TREE (Nguyen et al. 2015) with the best model (TVM + F + I) including 1000 replication of bootstrap analyses. The result showed that *M. ofeliae*, as a member of sect. *Talauma*, is a sister to all other magnolias in the subfamily Magnolioideae and the relationship is highly supported.

**Figure 1.**
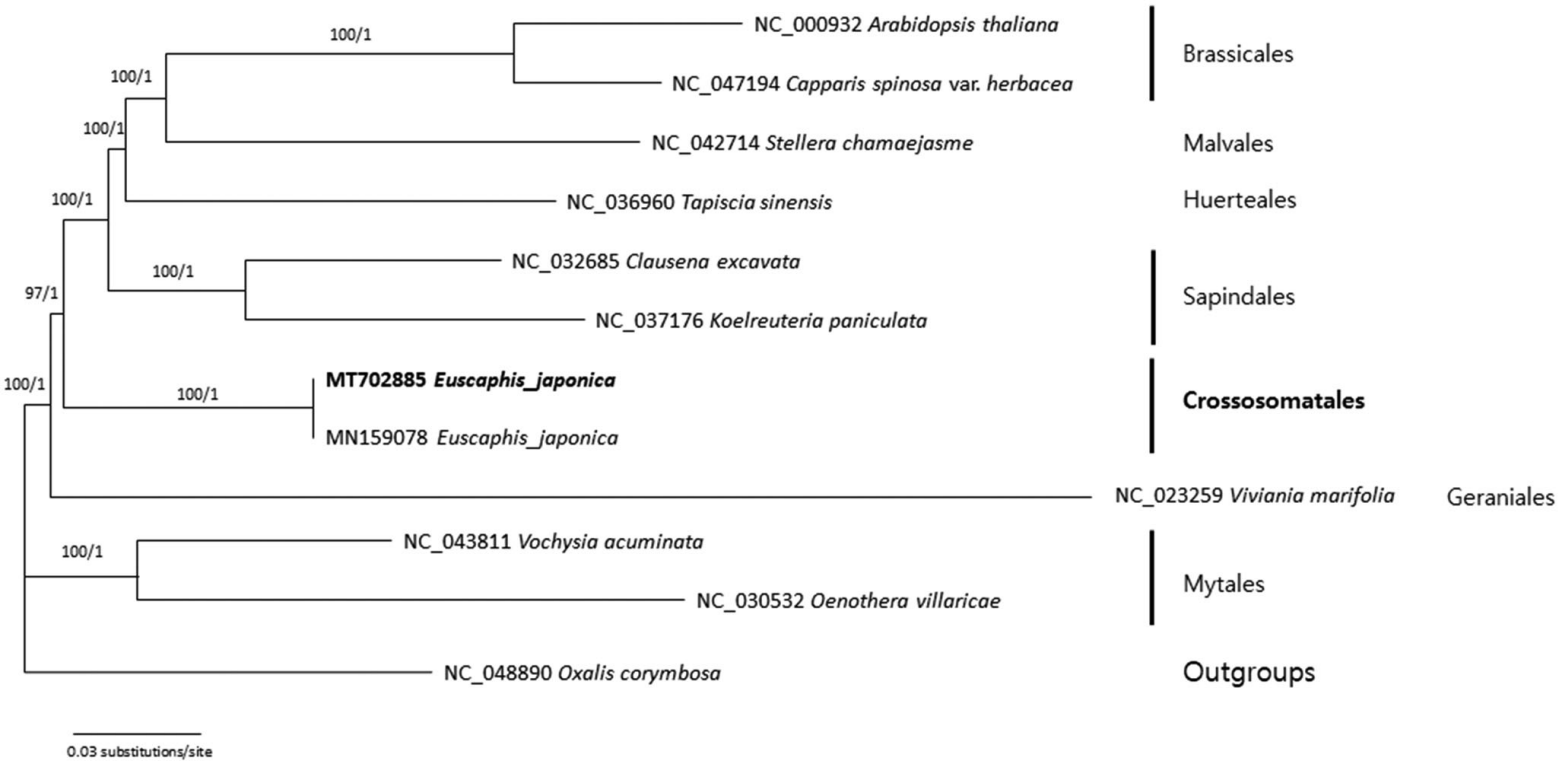
A maximum-likelihood tree based on an alignment of entire cp genome sequences that are from *M. ofeliae* and selected members of Magnoliaceae.

The cp genome of *M. ofeliae* reported in this study will play an important role in understanding the evolution and diversification of the family. Furthermore, it will be an important genetic resource to the studies of conservation and restoration of *M. ofeliae* as an endangered Mexican magnolia.

## Data Availability

The data that support the findings of this study are openly available in NCBI at https://www.ncbi.nlm.nih.gov/ (reference number: MT522025) or available from the corresponding author.
